# Management Strategies for *Helicobacter pylori*—Application of the Stewardship Principles

**DOI:** 10.1111/hel.70158

**Published:** 2026-10-13

**Authors:** Francis Megraud, Emilie Bessède, Tamara Matysiak‐Budnik

**Affiliations:** ^1^ INSERM U1312 BRIC Team 4 University of Bordeaux Bordeaux France; ^2^ IMAD, Hepato‐Gastroenterology & Digestive Oncology University Hospital Nantes France

**Keywords:** amoxicillin, antimicrobial susceptibility testing, bismuth quadruple therapy, clarithromycin, proton pump inhibitor, triple therapy

## Abstract

The management strategies for *Helicobacter pylori* treatment are still diverse, especially because of the unequal availability of the drugs needed. In addition, the development of resistance to some antibiotics leads to unsatisfactory results. The role of *H. pylori* in gastric cancer, which allows the possibility of prevention in the general population by eradicating the bacteria, is a stimulus to have well‐established regimens. This article presents the antimicrobial stewardship principles that can guarantee appropriate treatment. The optimization of the clarithromycin‐based triple therapy includes antimicrobial susceptibility testing for clarithromycin, a higher dose of amoxicillin than in the original regimen, more than twice a day, and a proton pump inhibitor (PPI) like rabeprazole or esomeprazole, all administered for 2 weeks. The other regimen often used, the bismuth quadruple therapy, is optimal with the 3‐in‐1 capsule and a standard or high dose of PPI. In all cases, an eradication control must be performed after 4 to 6 weeks. The so‐called concomitant treatment does not meet stewardship principles. The availability of Potassium‐Competitive Acid Blockers (P‐CABs) in some countries could allow the use of a dual therapy with only amoxicillin, but it must be confirmed by proper trials in Western countries.

## 1 Introduction

*Helicobacter pylori* infection is very common worldwide, and it has been recognized that almost half of the world population is infected [1]. The infection may lead to peptic ulcer disease, and the discovery of this causal relationship was rewarded with the Nobel Prize attributed to R. Warren and B. Marshall in 2005 [2]. Furthermore, the carcinogenic role of *H. pylori* , already mentioned in 1994 [3], was later confirmed, and it is currently estimated that over 90% of all cases of gastric cancer are related to chronic *H. pylori* infection. Gastric adenocarcinoma is indeed one of the major cancers of infectious origin, and globally it is in 5th position for morbidity and mortality by cancer [4].

This background of severe diseases, as well as other possible impacts on health, led to the consideration that all *H. pylori* should be eradicated [5] and programs should be developed for primary prevention of gastric cancer by *H. pylori* eradication in countries with a high incidence, *i.e*., in the range of 10 cases per 100,000 person‐years or more. For countries with low or medium incidence, only the population groups at high risk should be targeted [6]. However, this does not apply to children because, despite the fact that the infection is acquired in childhood, there are usually no clinical consequences at this age and, furthermore, the infection may contribute to the maturation of the immune system [7].

The aim of this article is to review the management strategies for *H. pylori* infection and to apply the stewardship principles to obtain the maximum efficacy, minimizing the adverse events and preserving the activity of antibiotics for the future.

A literature search was performed on PubMed.

These principles have been developed for more than 20 years to try to maintain the activity of current antimicrobials [8] and have been recently recommended in population‐based *H. pylori* screen and treat programs [9]. The stewardship principles already published in the context of *H. pylori* infection [10, 11, 12, 13] are updated in this review.

### 1.1 Historical Background on *Helicobacter pylori* Treatments

*H. pylori* treatments were developed, essentially empirically, in the 1990s. Following the limited effect of mono‐ and dual therapies, two triple therapies, including an antisecretory drug recently developed at that time: Omeprazole and either clarithromycin‐tinidazole [14] or clarithromycin‐amoxicillin [15], were presented at the VI^th^ Workshop of the European *Helicobacter Pylori* Study Group in Brussels in 1993. Both were given for 7 days and led to promising results. Then, a large randomized clinical trial was performed (MACH2 study), including 539 patients randomized into 4 groups: Clarithromycin (500 mg) and metronidazole (400 mg) or clarithromycin (500 mg) and amoxicillin (1 g); both with or without omeprazole (20 mg), all treatments administered twice a day for one week. The results presented in Table 1 show the value of the triple therapies, including proton pump inhibitors (PPIs), which were used extensively at that time under the name of French or Italian therapy [16].

**TABLE 1:** Rates of eradication expressed in % and obtained in the mACH2 study, which included 539 patients with a history of duodenal ulcer & *H. pylori* infection.

Therapy	ITT	PP
	(514)	(449)
AC	26%	25%
OAC	94%	95%
CM	69%	72%
OCM	87%	91%

Abbreviations: A, amoxicillin; C, clarithromycin; ITT, intention‐to‐treat; M, metronidazole; O, omeprazole; PP, per protocol.

In addition, another type of therapy was developed in parallel: The bismuth‐based quadruple therapy (BQT). It began with a bismuth‐based triple therapy including a bismuth salt with two antibiotics: Tetracycline and metronidazole [17]. The results appeared better by adding an anti‐secretory drug, first an H_2_ receptor antagonist, then a PPI such as omeprazole in 1995 [18]. In 1998, the final BQT was established by Borody et al. [19] and later a company sold the 3‐in‐1 capsule (bismuth salt, tetracycline, metronidazole) to which the PPI has to be added. This combination improves compliance.

Throughout the years, the development of clarithromycin resistance, mainly due to the common use of this antibiotic for respiratory infections, led to an increase in the length of the triple therapy from 7 to 10 or 14 days, and its use was even discouraged when the clarithromycin resistance in the area was > 15% [20]. However, *H. pylori* resistance did not concern the BQT because resistance to metronidazole, the only antibiotic included in this regimen for which resistance may occur, can be overcome by bismuth salts as well as by a higher dose and a longer treatment duration.

### 1.2 The Stewardship Principles of Eradication Treatments

As far as the stewardship principles for *H. pylori* eradication treatment are concerned, the ‘5D rule’ must be followed, which involves the choice of the drug, the dose to prescribe, the optimal drug route, the suitable duration, and the timely de‐escalation or control to obtain an eradication success of 90% or more, as in any other infection (Table 2). Regarding the drug to be used for the triple therapy, the problem is essentially related to antimicrobial resistance. The WHO proposed a global strategy for containment of antimicrobial resistance already 15 years ago [21], and in 2017 *H. pylori* was included in the priority list of pathogens for which new antibiotics were needed, due to its resistance to clarithromycin [22]. Indeed, clarithromycin is the most important antibiotic concerned by the resistance because of its frequent use. Resistance to levofloxacin, a fluoroquinolone used as a second‐line therapy, also has a high impact on the success of the therapy. In contrast, antimicrobial resistance to the other antibiotics (amoxicillin, tetracycline, rifabutin) is not so important because it is uncommon. Metronidazole is a special case because there is a lack of reproducibility of in vitro resistance data, and in vivo it has a limited impact on efficacy, probably because of a synergy with either the other antibiotic or bismuth.

**TABLE 2:** Antimicrobial stewardship principles: The ‘5d’ rule.

Right **D**rug
Correct **D**ose
Right **D**rug Route
Suitable **D**uration
Timely **D**e‐escalation

#### 1.2.1 The First Principle of Stewardship Is to Use the Right Drug

To fulfill the first principle of stewardship, it is necessary to perform antimicrobial susceptibility testing (AST) for clarithromycin. While in the past, culture with an antibiogram was the only method available, it is now possible to perform a real‐time PCR (RT‐PCR) directly on gastric biopsies, which allows the detection of both *H. pylori* and the mutations associated with clarithromycin resistance. There are two positions (2142, 2143) on the 23S rRNA where a mutation can occur, and they are easily detectable. A fluorescence resonance energy transfer (FRET) method can be used, and a melting curve analysis (MCA) shows the presence of the possible mutation [23]. There are a large number of kits currently available to perform this analysis directly on the gastric biopsies without the necessity to culture the strain. In addition, following the COVID pandemic, most laboratories are equipped with thermocyclers, allowing this analysis to be performed. This technique is rapid and cheap [24]. Because of this evolution, it can now be easily performed in clinical laboratories. There are other molecular methods available, such as sequencing of the PCR products, droplet digital PCR, and next‐generation sequencing [25, 26]. This last method is the most powerful, allowing an exploration of the mutations associated with all of the antibiotics, but at this stage, it is more a research tool than a day‐to‐day method, because it is long and expensive. It may help to better characterize amoxicillin and metronidazole resistance, for which our knowledge is not yet complete. In summary, it is mandatory to test clarithromycin susceptibility before prescribing this antibiotic in most of the European countries and also worldwide. In some countries where the clarithromycin resistance rate is < 15% [27], i.e., the limit for AST usually proposed in the guidelines, it is possible to avoid this test. However, it must be kept in mind that there is often variability between different areas of a given country.

The right drug, as mentioned in this first stewardship principle, also concerns the choice of the PPI because most are metabolized by the CYP‐2C19 hepatic enzyme in the so‐called rapid metabolizers. Only two PPIs are not subject to this degradation and therefore should be used for the triple therapy: Esomeprazole and rabeprazole [28]. Indeed, rabeprazole is mostly, although not exclusively, degraded via non‐enzymatic reduction, while esomeprazole acts as an inhibitor of CYP‐2C19, slowing its own degradation and thus increasing its bioavailability with repeated doses.

Another limit of this strategy is the need to obtain gastric biopsies, which necessitates an upper digestive endoscopy. Indeed, for young patients (< 45 y) who are not at risk of gastric cancer and do not have alarm symptoms, an endoscopy is not always necessary, and non‐invasive tests can be performed to diagnose *H. pylori* [20]. Then, there are two possibilities to perform this diagnosis: Either by (1) testing stools, but the low amount of *H. pylori* DNA requires an excellent extraction method even if positive results have been published [29], and the sensitivity of this method may not be sufficient, or (2) by testing gastric juice by swallowing a capsule attached to a highly absorbent nylon string to get the mucus present on the string after pulling it out, namely the string test [30]. This last method should be used more when a fiber optic endoscopy is not performed.

This first principle of stewardship is in favor of abandoning the so‐called concomitant therapy, which includes three antibiotics given simultaneously: Clarithromycin, amoxicillin, and metronidazole, in addition to a PPI. Indeed, if there is clarithromycin susceptibility, a third antibiotic is not needed, which avoids the use of huge quantities of unnecessary antibiotics and limits the negative impact on the gut microbiota [31, 32]. However, the recommendations, which date back several years, did not yet include this point. In this respect, it is interesting to compare the Toronto consensus published in 2016 [33], which considered that one of the treatment options was a 14‐day concomitant treatment, in spite of moderate evidence, with the ACG clinical guidelines published in 2024 [34] that did not recommend this treatment. During the period between the two guidelines, antimicrobial resistance was increasing, and the stewardship principles were highlighted.

With regard to the BQT, there is no problem of resistance given that tetracycline resistance is very rare and metronidazole resistance is surpassed by the combination. A PPI, e.g., omeprazole, must be prescribed at a standard dose twice a day.

#### 1.2.2 The Second Principle of Stewardship Is to Use a Correct Dose and Dose Fractionation

Again, this point concerns the triple therapy and especially amoxicillin. It turns out that the 2 g/day dose originally proposed is not sufficient for most patients [35]. The minimum should be 3 g/day and even more for those with a high body mass index (BMI), i.e., 50 mg/kg/day. Another problem with the original regimen is the frequency of drug administration because administering amoxicillin twice a day is not sufficient. Indeed, in contrast to clarithromycin, amoxicillin does not penetrate into the epithelial cells but passes between the cells to reach the gastric mucus, so this diffusion occurs only when there is amoxicillin in the blood [36]. For this reason, the optimal administration is 3 to 4 times a day. It is also important to prescribe a high dose of PPI (potency of acid inhibition 54–128 omeprazole equivalent) together with amoxicillin to increase the gastric pH as much as possible, which favors the activity of the antibiotics.

For the BQT, optimal effectiveness was obtained with a high dose of PPI in 10‐ and 14‐day treatment, while a high or standard dose (potency of acid inhibition 32–40 omeprazole equivalent) was sufficient for second‐line, all given twice a day [37].

#### 1.2.3 The Third Principle of Stewardship Is to Use the Right Drug Route

Given that *H. pylori* infection is essentially treated at home and not at the hospital, the oral route is used. In very rare cases, an IV treatment is needed. A systematic review was published presenting the results of 11 studies with IV treatment but without robust trials. Nevertheless, an IV route appears to be a feasible therapeutic alternative for patients where oral therapy is contraindicated [38].

It is of interest to administer the PPI about an hour before the meal to have a better impact on gastric acidity and then the antibiotics during the meal.

#### 1.2.4 The Fourth Principle of Stewardship Is to Apply a Suitable Duration of Treatment

For the triple therapy, the treatment duration has to be optimized. The 7‐day duration proposed at the beginning does not lead to the best results. A 10‐day duration is preferable; however, the best results are obtained with 14 days of treatment, and this duration is currently recommended, without major negative consequences on adverse events [20].

For the BQT, a 14‐day duration is also used; however, in the case of the availability of the 3‐in‐1 capsule (Pylera^R^), 10 days appear to be sufficient [39]. The cause of the slightly better results obtained with the 3‐in‐1 capsule could be due to the fact that it is more practical for the patient, leading to better compliance. Unfortunately, Pylera^R^ is not yet available in many countries, but its availability is progressively increasing. It is important to note that the PPI dose to be used in BQT is at least 40 mg of omeprazole twice a day.

In all cases, an important recommendation for the physician is to explain the possible adverse events and the need to pursue the treatment to the patient and, if possible, by giving him/her written information.

#### 1.2.5 The Last Stewardship Principle, i.e., Using a Timely De‐Escalation

Strictly speaking this last principle is not relevant to *H. pylori* treatments which are short term. However, the choice of a triple therapy after AST instead of a BQT could be considered a de‐escalation, which avoids unnecessary drugs.

The need to perform a test of cure is necessary four to six weeks after the end of the treatment, and more importantly, at least two weeks after stopping the PPI.

This control can be performed using the urea breath test (UBT), a well‐standardized, non‐invasive, and easy‐to‐perform test [40]. Another possibility is to use the stool antigen test (SAT) with monoclonal antibodies and an ELISA protocol, although it is in the range of 10% less sensitive [41].

In summary, the current recommendations for *H. pylori* treatment are presented in Table 3.

**TABLE 3:** Current recommendations for *Helicobacter pylori* treatment, allowing an eradication rate in the range of 90%.

**Triple therapy**
Esomeprazole or Rabeprazole 2 × 20 mg
Clarithromycin 2 × 500 mg
Amoxicillin 3 × 1 g minimum (50 mg/kg for heavy patients)
Per day for 14 days
**Bismuth Quadruple Therapy**
Pylera^R^: Four capsules 3 times a day (after meals) for 10 days or three capsules 4 times a day (after meals and at bedtime) for 10 days
Plus Omeprazole 20 mg twice a day for 10 days or an equivalent PPI
Each Pylera^R^ capsule contains bismuth sub‐citrate 140 mg, tetracycline 125 mg, and metronidazole 125 mg.
These 3 components can also be prescribed separately if Pylera^R^ is not available as it is in several countries, but the recommendation is then for 14 days.
***p*‐CAB dual therapy**
The *p*‐CAB mostly used is Vonoprazan 2 × 20 mg per day for 14 days
The usual antibiotic combined is Amoxicillin given 3 × 1 g minimum (50 mg/kg for heavy patients) per day for 14 days
This treatment is currently not available in Europe except for compassionate use

### 1.3 Other Points to Consider

#### 1.3.1 New Antisecretory Drugs

These last years, a new type of antisecretory drugs has been developed: Potassium‐competitive acid blockers (P‐CABs), especially with the first one available, vonoprazan. These compounds have a quicker and more intense activity than PPIs on the gastric pH [42], and could allow the physician to prescribe only one antibiotic, such as amoxicillin, for which resistance is uncommon, and so AST could be eliminated. This treatment is indeed the ultimate proposal to fulfill the stewardship principles. Vonoprazan‐amoxicillin dual therapies have been used in Asian countries with good results [43]. A randomized controlled trial (RCT) was performed in the US and Europe to test this dual therapy as well as a triple therapy, vonoprazan‐amoxicillin‐clarithromycin, in comparison to a PPI‐based triple therapy, but the results of this trial were not those which were expected for the P‐CABs [44]. Indeed, the drug dosages were based on those used in Asian populations, which is probably not optimal for Westerners. There is a need to optimize this regimen, especially taking into account the higher BMI in Western populations. Furthermore, P‐CABs are still not available in several areas of the world, such as Europe.

To be in line with the stewardship principles, only P‐CAB dual therapies should be prescribed [45].

#### 1.3.2 Adjuvant Therapy

The only adjuvant therapy to consider is a probiotic, and the essential benefit of adding a probiotic is to increase compliance, despite the necessity to prescribe additional drugs to a therapy that is already comprised of many pills. However, the results show a positive impact with better compliance and higher eradication rates. Lactobacilli could be used, but the best results are obtained with *Saccharomyces boulardii*, which is resistant to all antibiotics used in the treatment [46]. Even P‐CAB dual therapies can benefit from the addition of *S. boulardii* [47].

#### 1.3.3 Allergic Patients

As triple therapies include amoxicillin, a beta‐lactam antibiotic, it cannot be used if the patient is allergic to such compounds. A possibility is to replace it with metronidazole. The triple therapy PPI‐clarithromycin‐metronidazole is indeed one of the original triple therapies. However, allergy to beta‐lactams is probably less frequent than we think and deserves to be explored by an allergist; if necessary, a desensitization can be carried out.

#### 1.3.4 Patients' Comorbidities

Another issue may concern different comorbidities, like renal insufficiency or some heart conditions (long QT syndrome), that may limit the use of certain antibiotics. In these specific situations, a case‐by‐case approach, involving respective specialists, should be considered, and antibiotic testing is particularly important to choose the most appropriate drug in terms of its efficacy and potential toxicity.

The question of stopping attempts to eradicate *H. pylori* after several failures must also be considered. The benefits vs. disadvantages should be taken into account according to the patient's age and existing comorbidities. Eradication treatment in *H. pylori* ‐positive gastric MALT lymphomas, considered as the first‐line treatment of lymphoma, is strongly indicated and should be pursued until effective eradication. The presence of a peptic ulcer and the risk of bleeding also remain reasons for pursuing the goal. In such cases, for patients taking medication, it is important to consider the possible drug–drug interactions. It is also important to adapt the dosage in case of chronic kidney disease or chronic liver disease.

If the precancerous lesions eventually present are not too advanced in an elderly patient already taking various drugs, it may be preferable to abstain and perform surveillance.

#### 1.3.5 Countries With Limited Availability of Drugs or Testing

In most of these countries, there is a high resistance rate to many of the antibiotics, especially clarithromycin, so it should be avoided in the treatment. Levofloxacin is not recommended either because of its potential rare but severe adverse events. If any bismuth salt is available, it could be added to two other antibiotics present on the market, e.g., tetracycline and metronidazole (or tinidazole).

Concerning AST, culture and antibiogram testing, although long and demanding, remain a possibility in all countries.

Our review will not go into detail on the second treatment in case of failure of the first one, given that if the first‐line treatment is carried out properly, only a very limited number of patients will still be *H. pylori* positive. Many guidelines recommend the use of BQT. If another triple therapy has to be prescribed, it is important not to use clarithromycin again but to replace it with either levofloxacin, which necessitates AST, or rifabutin.

## 2 Conclusion

The main stewardship principles for *H. pylori* treatment include the use of the right antibiotic, at the right dose and for the right duration, insisting on good patient compliance, which is another important factor of treatment success.

If a triple therapy is planned, clarithromycin resistance must always be tested, which is possible and easy by RT‐PCR. The dose of amoxicillin should be optimized, and this antibiotic should be prescribed more than twice a day for 14 days, using esomeprazole or rabeprazole as the antisecretory drug. The future is in favor of a dual therapy using a P‐CAB, e.g., vonoprazan, with a high dose of amoxicillin given more than twice a day, but RCTs are needed to confirm the optimal dosage to be used.

## Author Contributions

Discussion of the topic and agreement on a frame (FM, EB, TM), writing of the 1st version (FM), review and corrections (EB, TM).

## Funding

The authors have nothing to report.

## Disclosure

Artificial intelligence was not used to prepare this manuscript.

## Conflicts of Interest

The authors declare no conflicts of interest.

## Data Availability

The data that support the findings of this study are available from the corresponding author upon reasonable request.
